# Long-term impacts of parental migration on Chinese children’s psychosocial well-being: mitigating and exacerbating factors

**DOI:** 10.1007/s00127-017-1386-9

**Published:** 2017-04-24

**Authors:** Chenyue Zhao, Feng Wang, Leah Li, Xudong Zhou, Therese Hesketh

**Affiliations:** 1 Institute for Global Health, University College London, 30 Guilford St, London, WC1N 1EH UK; 2 Institute of Social Medicine, Zhejiang University, 866 Yuhangtang Rd, Hangzhou, 310058 Zhejiang China; 3 Population, Policy and Practice Programme, GOS Institute of Child Health, University College London, Guilford St, London, WC1N 1EH UK; 4 Centre for Global Health, Zhejiang University School of Medicine, 866 Yuhangtang Rd, Hangzhou, 310058 Zhejiang China

**Keywords:** Migration, Left-behind children, Child well-being, Family relations, Social support

## Abstract

**Purpose:**

Prolonged separation from migrant parents raises concerns for the well-being of 60 million left behind children (LBC) in rural China. This study aimed to investigate the impact of current and previous parental migration on child psychosocial well-being, with a focus on emotional and behavioral outcomes, while considering factors in family care and support.

**Methods:**

Children were recruited from schools in migrant-sending rural areas in Zhejiang and Guizhou provinces by random stratified sampling. A self-administered questionnaire measured children’s psychosocial well-being, demographics, household characteristics, and social support. Multiple linear regression models examined the effects of parental migration and other factors on psychosocial difficulties.

**Results:**

Data from 1930 current, 907 previous, and 701 never LBC were included (mean age 12.4, SD 2.1). Adjusted models showed both previous and current parental migration was associated with significantly higher overall psychosocial difficulties, involving aspects of emotion, conduct, peer relationships, hyperactivity, and pro-social behaviors. Parental divorce and lack of available support demonstrated a strong association with greater total difficulties. While children in Guizhou had much worse psychosocial outcomes than those in Zhejiang, adjusted subgroup analysis showed similar magnitude of between-province disparities regardless of parental migration status. However, having divorced parents and lack of support were greater psychosocial risk factors for current and previous-LBC than for never LBC.

**Conclusions:**

Parental migration has an independent, long-lasting adverse effect on children. Psychosocial well-being of LBC depends more on the relationship bonds between nuclear family members and the availability of support, rather than socioeconomic status.

## Introduction

Increasing migrant flows in many parts of the world are leading to prolonged separation of family members on an unprecedented scale. The number of these so-called “left-behind children” (LBC) is high in many low- and middle-income countries [1, 2]. Historically migration has more often involved young people prior to having families. However, patterns have now changed in many parts of the world as the effects of globalization, ease of movement, and the economic incentives have encouraged and facilitated huge migrant flows. Much of this migration occurs from rural to urban areas within countries for work, often on a temporary basis. China is a typical case where massive rural–urban migration has resulted in 61 million children being left behind in rural areas, accounting for 38% of rural children and 22% of total child population in China [3].

For parents, the decision to migrate involves a trade-off: parents working away may contribute to increased family income and better education opportunities for children, yet lack of parental monitoring, supervision, and support may result in a range of psychosocial and developmental risks [4]. Child psychology and family study researchers have delineated frameworks of child psychopathology in relation to prolonged absence of parents or caregivers, especially within the ambit of attachment theory [5–7]. Factors that contribute to attachment relationships include the presence of the caregiver, duration and quality of care, and emotional investment [6]. During long periods of separation, older children may develop negative emotions and dysfunctional thoughts similar to young children’s reactions to physical separation [7].

Concerns about the impacts of being left behind by migrant parents have also led to a growing body of empirical research in child psychology. Parental migration is found to be associated with loneliness [8–10], low self-esteem [11], depressive and anxiety symptoms [11–14], risk behaviors [15], poor school performance and early dropout [15–17]. Studies have reported that migration type (father-only, mother-only, or both-parent migration) and care arrangements [18–20], as well as child age [21, 22] and sex [15, 23] were important correlates of LBC’s mental health outcomes. However, most of the literature on LBC’s psychological well-being does not provide in-depth exploration of household characteristics and dynamic family relationships. These factors, such as parental divorce and family and social support, may complicate the effects of prolonged separation from parents. Analyses on how the parent–child relationship is experienced within the constraints of physical separation should take into account the dynamism and complexity of the family ties [24]. Across migrant-sending communities in many countries, managing the impact of migration on the vulnerable left-behind family members has become a major challenge, from economic, social, family, and individual perspectives [25].

Separation and reunion in migrant families may also take various patterns [26]. In China, since the vast majority of migration flows are within the country, it is not uncommon that migrant parents return home after living apart from their child for an extended period. There has been very limited research into the effects on children after the return of parents [17, 21]. Changes in China’s labor market with declines in the manufacturing sector and the shift of companies to rural areas have meant that more parents are returning after prolonged periods away from home [27].

The overall aims of this study are to investigate (1) the impact of parental migration on the psychosocial well-being of children who are currently left behind, in comparison with those who were previously left behind, (2) how child well-being factors within the child’s family and social environments, such as care provision, psychosocial support, and socioeconomic status, may affect the impact of parental migration, and (3) whether the effects of key psychosocial risk factors are greater in left-behind children than never left-behind children.

## Methods

### Participants

Participants were recruited from migrant-sending rural areas in Zhejiang and Guizhou Provinces, China. Three counties in western Zhejiang (Kaihua, Jiande, and Jiangshan) and two counties in southeast Guizhou Province (Guiding and Longli) were included in the study. Zhejiang is a wealthy coastal province, but its western mountainous area is relatively underdeveloped; Guizhou is one of the poorest provinces in China, although the two counties included in this study rank at medium level in GDP across the province.

Questionnaires were obtained from 3596 children. Nine children who did not complete the SDQ section, and eight children with one or both parents deceased were excluded. Another 41 (1.1%) children failed to report parental migration status. A total of 3538 participants were included in the analysis, comprising 1930 current-LBC, 907 previous-LBC, and 701 never-LBC.

Mean age of the sample was 12.4 (SD 2.1) with previous-LBC slightly older (mean 12.5, SD 2.1) than the other two groups. Overall, there were more girls (52.5%) than boys (47.5%) and this did not differ across the three groups. In our sample, 61.8% children were recruited in Guizhou and 31.2% in Zhejiang, and a higher proportion of children in Guizhou (65.6%) were ever left-behind.

### Procedure

Officials at the local governments were interviewed to understand the socioeconomic contexts and identify communities with high levels of out-migration. For inclusion in the study, twenty migrant-sending townships were selected in Zhejiang, and ten were selected in Guizhou. In Zhejiang, to make the sample more comparable to Guizhou, two villages at lower economic development levels in each township were further chosen as an inclusion criterion. In both provinces, two random schools in each selected township were included in the study, and all students present in these schools at the time of survey, from Year 4 to Year 9 (aged 9–17), were selected; and then, in Zhejiang, only students from the selected poorer villages were included in the final sample.

This study was approved by the Ethics Committees of University College London and Zhejiang University. Participants were guaranteed the anonymity and confidentiality of their responses in the questionnaire. Before the survey, both the eligible student and one of their parents or custodial caregivers (usually a literate grandparents) were provided with an informed consent form enclosing a detailed description of this study. If consent was given on both forms, the child was asked to complete a self-administered questionnaire in their classroom, without the presence of any teachers or school administrators. Participants were told that there was no compulsion to complete the questionnaire, even after consent was obtained.

### Measures

Child psychosocial well-being was measured with the self-report version of Strengths and Difficulties Questionnaire (SDQ) [28]. The SDQ comprises 25 items of psychosocial well-being in five dimensions, including emotional symptoms, conduct problems, hyperactivity, peer problems, and pro-social behaviors. Each item was scored from 0 to 2. Each dimension was measured by the summed score of its five items as a subscale. A total difficulties score, ranging from 0 to 40, was derived as the sum of four subscales (excluding pro-social subscale), with higher scores indicating greater difficulties. The SDQ has proven its reliability and validity across different cultures and settings, and has been validated in Chinese population [29].

Parental migration status was determined according to the two questions “has your father (and mother) taken a job away from your hometown and been absent for over six months?” The options were “yes, currently absent”, “yes, previously absent”, and “no, never”. If one or both parents were currently absent, the child was defined as a “current-LBC”; if not, and if one or both parents were previously absent, the child was defined as “previous-LBC”; and if neither parent was ever away, the child was “never-LBC”.

Household characteristics, including economic status, parents’ marriage status, primary caregiver, and siblings, were assessed. Economic status was measured by the number of household appliances, including air conditioner, refrigerator, washing machine, television, and computer. The variable was then coded as poor (zero to one item), fair (two to three items), and wealthy (four to five items). The primary caregiver of children was identified based on two consecutive questions, “Who are you currently living with?” and, “Among them, who takes care of you the most?” Caregivers were grouped into four categories: grandparents, father, mother, and others (including relatives, friends, siblings, and no caregiver).

Social support was measured by a scale of six items adapted from the Multidimensional Scale of Perceived Social Support [30]. We focused on the aspects that better apply to rural children in China. The questions asked were: whether or not there is someone who the child could turn to help (1) for difficulties in studying, (2) for personal problems, (3) if the child was teased or bullied, (4) if they were feeling sad or depressed, (5) for guidance in dealing with issues when things go wrong, (6) to share happiness with. Children who scored 6, i.e., answered “yes” in all six items, were categorized as high social support. Children who scored 3–5 were categorized as medium, and 0–2 as low social support.

School performance was measured by the question, “In general, what is your academic performance level in your class?” The options were “top, upper-middle, middle, lower-middle, low”, coded from 1 (low) to 5 (top). As in most of China, students are informed of their ranking in the class for major tests, so all children are able to answer this question without difficulty.

### Statistical analysis

Chi-square test and analyses of variance were conducted to compare sample characteristics, across three groups of children with different parental migration status. Multiple linear regression models were applied to examine the associations between the psychosocial outcomes and the parental migration status. The initial model included child demographics (age, gender, and province). The model was further adjusted for household characteristics, including economic status, caregiver and sibling, and parents’ marriage status, and two other covariates, social support and school performance. For covariates that remained significant (i.e., *p* ≤ 0.05), their interactions with migration status were tested, within the adjusted model. Then, the adjusted cell means of total difficulties score were estimated, treating each level of the interaction term as balanced (equal group size), and compared pairwise.

## Results

Table 1 presents the descriptive statistics of children by their parental migration status.

**Table 1:** Sample characteristics by parental migration status

	Current-LBC	Previous-LBC	Never-LBC	*p* value
	Mean/N (SD/%)	Mean/N (SD/%)	Mean/N (SD/%)	
Primary caregiver				<0.001
Grandparent	1254 (65.2)	252 (27.9)	124 (17.8)	
Father	150 (7.8)	147 (16.2)	138 (19.8)	
Mother	390 (20.3)	486 (53.7)	431 (61.8)	
Other	128 (6.7)	20 (2.2)	5 (0.7)	
Parents divorced				<0.001
No	1637 (85.5)	812 (90.3)	665 (95.6)	
Yes	278 (14.5)	87 (9.7)	31 (4.5)	
Any sibling				0.064
No	434 (22.5)	170 (18.8)	157 (22.4)	
Yes	1494 (77.5)	736 (81.2)	544 (77.6)	
Household wealth				<0.001
Poor	185 (9.6)	62 (6.8)	53 (7.6)	
Fair	1181 (61.3)	564 (62.3)	291 (41.5)	
Wealthy	562 (29.2)	280 (30.9)	357 (50.9)	
Social support				0.003
Low	122 (6.4)	38 (4.3)	38 (5.5)	
Fair	780 (40.8)	346 (38.8)	238 (34.2)	
High	1010 (52.8)	507 (56.9)	420 (60.3)	
School performance				0.017
Very poor	155 (8.1)	77 (8.5)	58 (8.3)	
Poor	375 (19.5)	167 (18.5)	117 (16.7)	
Fair	849 (44.2)	390 (43.1)	277 (39.6)	
Good	400 (20.8)	188 (20.8)	164 (23.4)	
Very good	144 (7.5)	83 (9.2)	84 (12.0)	
Total difficulties score	13.1 (5.1)	13.3 (5.0)	12.3 (4.7)	<0.001
Emotional symptoms	3.9 (2.2)	3.9 (2.2)	3.5 (2.1)	<0.001
Conduct problems	2.3 (1.5)	2.4 (1.6)	2.2 (1.5)	0.130
Peer problems	2.9 (1.7)	3.0 (1.7)	2.7 (1.7)	<0.001
Hyperactivity	4.0 (2.0)	4.0 (1.9)	3.8 (1.9)	0.021
Pro-social behaviors	7.2 (1.9)	7.2 (1.9)	7.1 (1.9)	0.750

Means of age and SDQ scores are compared between groups by *F* tests. All other variables are categorical, and groups were compared by Chi-square tests

Nearly, two-thirds of current-LBC were primarily cared by grandparents, whereas respective proportions for previous-LBC and never-LBC were 28 and 18%. Parents who had migrated were more likely to be divorced. Current-LBC’s parents were about three times, and previous-LBC’s parents two times more likely than never-LBC’s parents to be divorced. Approximately one-fifth of the children were only children, across the three groups. Fifty percent of never-LBC reported that they were from wealthier households, compared with 30% from the two left-behind groups. In fact, currently-LBC were significantly poorer than never-LBC in both Zhejiang and Guizhou provinces (data not shown). Current-LBC also had lower social support (*p* < 0.001) and school performance (*p* < 0.001) compared to never-LBC.

Table 1 also shows the observed differences in mean total difficulties and subscale scores from SDQ, between the three groups of children. Both current-LBC and previous-LBC had higher mean scores of total psychosocial difficulties, as well as higher mean subscale scores of emotional symptoms, peer relationship problems, and hyperactivity, as compared to never-LBC. No differences were identified between the previous-LBC and current-LBC in total or subscale scores according to post hoc tests.

Multiple regression results demonstrated that parental migration, both previous and current, was associated with significantly higher scores in psychosocial difficulties (Table 2). Compared to never-LBC, increases in total difficulties score in current-LBC remained significant (*B* = 0.57, *p* = 0.017), after adjusting for household characteristics, social support, and school performance. The strong impact of previous parental migration changed little from the baseline model (*B* = 0.81, *p* = 0.001) to the adjusted model (*B* = 0.75, *p* = 0.003). Boys appeared to be marginally more vulnerable but only in the baseline model (*B* = 0.67, *p* = 0.051). Children in Guizhou had markedly higher psychosocial difficulties than those in Zhejiang (*B* = 0.92, *p* < 0.001). Age, household wealth, primary caregiver, and presence of siblings did not affect the psychosocial outcome. Parental divorce showed a strong association with higher total difficulties after adjusting all covariates (*B* = 1.00, *P* < 0.001). Both social support and school performances showed negative associations with total difficulties score; greater effects were indicated for each level decrease in social support and school performance. In addition, another adjusted model (not shown), which only included current LBC, indicated that child age at migrant parents’ initial departure as a continuous variable was strongly linked with worse well-being (*B* = −0.11, *p* = 0.001).

**Table 2:** Regression coefficient (SE) for total difficulties on parental migration status with and without adjustment for household characteristics, social support, and school performance

	Baseline model		Adjusted model	
	B	SE	B	SE
Parental migration				
PLB	0.81**	0.25	0.75**	0.25
CLB	0.75**	0.22	0.57*	0.24
Sex (ref: male)				
Female	−0.33	0.17	0.29	0.17
Age	0.02	0.04	0.03	0.04
Province (ref: Zhejiang)				
Guizhou	0.87***	0.18	0.92***	0.21
Household wealth (ref: fair)				
Poor			0.46	0.30
Wealthy			0.28	0.21
Primary caregiver (ref: Grandparent)				
Father			0.23	0.28
Mother			0.33	0.21
Other			0.29	0.42
Divorced parents			1.00***	0.27
Any Sibling			0.11	0.21
Social support (ref: high)				
Low			3.03***	0.37
Medium			1.32***	0.18
School performance (ref: fair)				
Very poor			2.66***	0.32
Poor			0.88***	0.23
Good			−0.90***	0.22
Very good			−1.39***	0.30

* *p* < 0.05; ** *p* < 0.01; *** *p* < 0.001

Table 3 presents the regression results of SDQ subscale scores that showed significant between-group differences in Table 1. After adjusting for all covariates, both current and previous absence of parents were still linked to emotional symptoms, whereas previous-LBC, rather than current-LBC, seemed more disadvantaged in terms of peer relationship, as compared to never-LBC. The demographic variables showed distinct patterns of associations with different subscale outcomes. Girls were much more likely to have emotional difficulties, whereas boys were more susceptible to peer relationship problems. Older children tended to have less peer relationship problems, but were more hyperactive, compared to younger children. Between the two provinces, only hyperactivity score did not differ between children from the two provinces. Although household wealth was negatively associated with peer relationship difficulties, children from richer families are more likely to have hyperactivity problems.

**Table 3:** Regression coefficients for emotional symptoms, peer relationships, and hyperactivity on parental migration status, household characteristics, social support, and school performance

	Emotional symptoms		Peer problems		Hyperactivities	
	B	SE	B	SE	B	SE
Parental migration status (ref: NLB)						
PLB	0.29*	0.11	0.19*	0.08	0.15	0.10
CLB	0.31**	0.11	0.09	0.08	0.16	0.09
Sex (ref: male)						
Female	0.84***	0.08	−0.12*	0.06	−0.07	0.07
Age	0.01	0.02	−0.11***	0.01	0.15***	0.02
Province (ref: Zhejiang)						
Guizhou	0.4***	0.09	0.19**	0.07	0.13	0.08
Household wealth (ref: fair)						
Poor	0.2	0.14	0.35**	0.10	−0.1	0.12
Wealthy	0.12	0.09	−0.17*	0.07	0.21*	0.08
Primary caregiver (ref: Grandparent)						
Father	−0.14	0.09	0.00	0.07	−0.08	0.08
Mother	−0.09	0.12	0.04	0.09	−0.02	0.11
Other	0.11	0.19	−0.21	0.15	0.08	0.17
Divorced parents	0.34**	0.12	0.17	0.09	0.25*	0.11
Any sibling	0.02	0.10	0.00	0.07	0.03	0.08
Social support (ref: high)						
Low	0.88***	0.16	1.14***	0.12	0.64***	0.14
Medium	0.39***	0.08	0.41***	0.06	0.44***	0.07
School performance (ref: fair)						
Very poor	0.55***	0.14	0.57***	0.11	0.83***	0.12
Poor	0.08	0.10	0.29***	0.08	0.32***	0.09
Good	−0.18	0.10	−0.19**	0.07	−0.4***	0.08
Very good	−0.21	0.13	−0.14	0.10	−0.88***	0.12

* *p* < 0.05; ** *p* < 0.01; *** *p* < 0.001

**Fig. 1:**
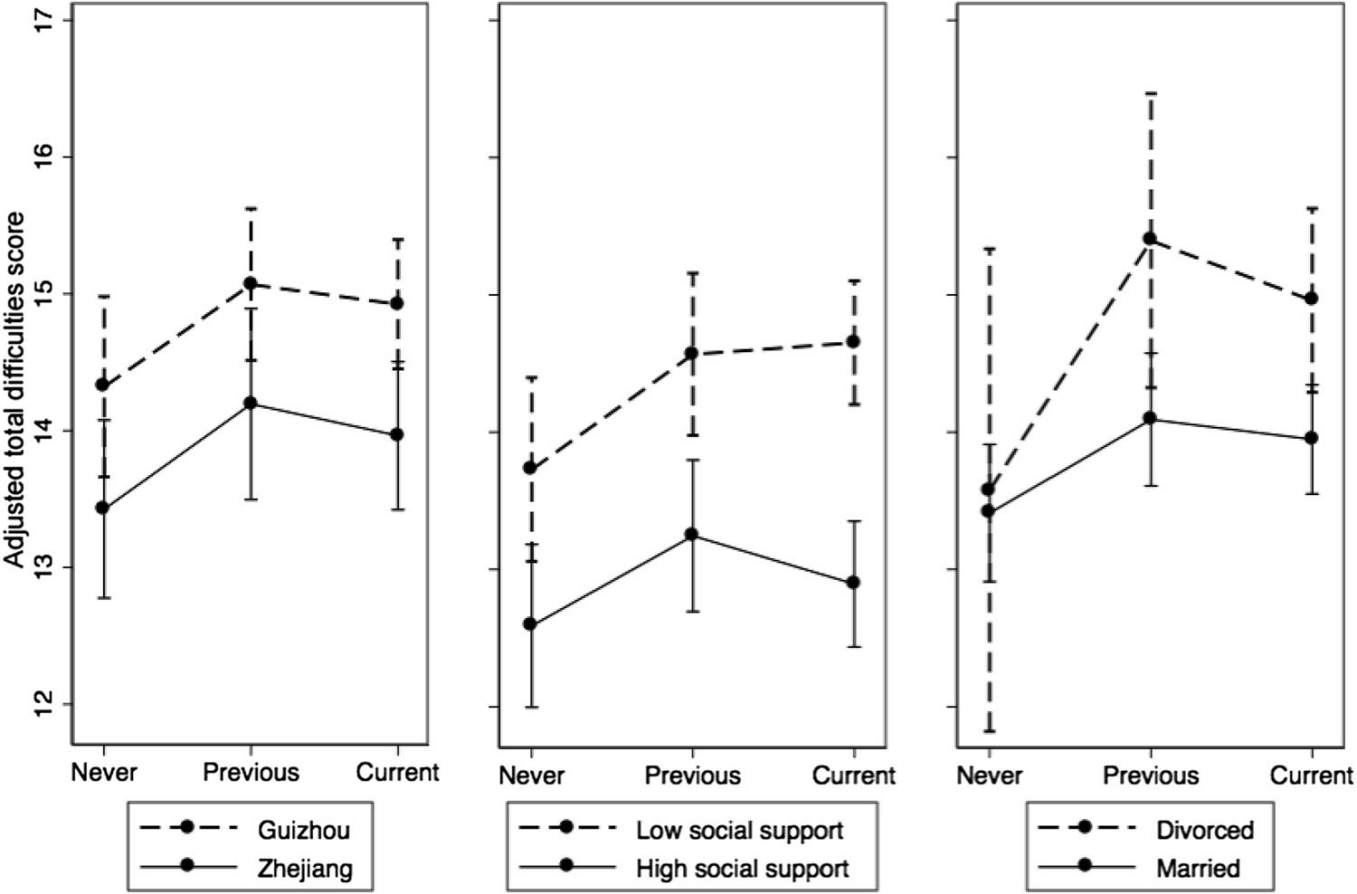
Adjusted total difficulties score by parental migration status and covariates

No significant interaction effects of parental migration and key covariates on psychosocial difficulties were found (data not shown). The magnitudes of the impact from parental migration on psychosocial difficulties were similar for Zhejiang and Guizhou (Fig. 1), despite the considerable child well-being gaps between the two provinces. However, the impacts of parents’ divorce and lower social support appeared to be greater in the two left-behind groups than the never left-behind group when comparing adjusted total difficulties scores between the divorced and undivorced and between low and high support groups, within each type of parental migration status (Table 4; Fig. 1).

**Table 4:** Between-subgroup comparisons of adjusted mean total difficulties score by key covariates and parental migration status

	Never-LBC			Previous-LBC			Current-LBC		
	Adjusted mean	*F*	*p*	Adjusted mean	*F*	*p*	Adjusted mean	*F*	*p*
Province		5.55	0.019		5.65	0.018		13.44	<0.001
Zhejiang	13.4			14.2			13.9		
Guizhou	14.3			15.0			14.9		
Divorce		0.03	0.854		5.58	0.018		9.88	0.002
Divorced	13.5			15.4			14.9		
Married	13.4			14.1			13.9		
Social support		9.04	0.003		16.01	<0.001		59.11	<0.001
Low support	13.7			14.5			14.6		
High Support	12.6			13.2			12.9		

## Discussion

Based on a sample selected from two Chinese provinces with widely differing socioeconomic development levels, our findings indicate that parental migration is independently associated with poor psychosocial well-being in children, especially emotional symptoms, peer relationship problems, and hyperactivity. The questionnaire survey achieved a high response rate, and collected detailed information about current and previous experiences of being left behind, as well as a range of child well-being correlates that allowed us to explore the characteristics of the child’s family and social environments.

Importantly, our results suggest that previous experience of prolonged separation from migrant parents had similar negative impacts on child well-being to the current absence of parents, after accounting for other factors. Adverse experiences related to parental absence in early childhood, such as disruption of parent–child communication and attachment relationship, have long-term negative psychological impacts [7]. The return of migrant parents is unlikely to reverse the complex consequences of their prolonged absence in the child’s life. Their return might even create new challenges in the child’s life due to the change of primary caregiver [31, 32]. However, further investigations are needed to elaborate the risks faced specifically by previous-LBC.

While children in Guizhou fared much worse than those in Zhejiang, our results demonstrated consistent impacts of parental migration on children across the two provinces. Household wealth did not correlate with children’s overall psychosocial outcome. These results suggest that better socioeconomic situation does not mitigate against the adverse psychological experiences caused by parental migration. In the poor rural areas, non-material support and care for left-behind children is probably as important for their well-being as the economic resources from remittances. In fact, wealthier migrant parents especially those who have obtained urban citizenship are more likely to take their children to the cities [33]. In recent years, there has been a marked increase of migrant children living in urban China, whereas the number of LBC remained largely stable in rural areas [34]. According to our findings, LBC also live in poorer families than other rural children. In other words, many children at risk of economic deprivation are separated from parents, and become susceptible to additional risks of psychosocial disadvantages.

Strong negative influences of parents’ divorce suggest the importance of supportive family dynamics in children’s mental health [35]. Parents’ divorce weakens the nuclear family bonds and diminished availability of the family as a supportive structure for the child [36]. Meanwhile, the composition of left-behind families did not seem to be an important factor; even when parents are at home, many children still see grandparents as primary carers. It is the availability of support, from either family or social environments, that plays an essential role in child well-being.

A crucial finding of this study is that the impacts of parental divorce and social support may be greater on left-behind children than never left-behind children. As China’s crude divorce rate has doubled during the past decade [37], with migration as a possible contributor, the consequences of parental divorce in LBC are particularly concerning. In addition to the disrupted parent–child relationship due to migration, parental divorce may cause further damage to the family dynamics for providing adequate care, and lead to extra adverse effects on children’s well-being. Lack of family and social support may result in huge challenges for the psychosocial development of children who are already deprived of parental care. Hence, children who are at these additional risks, besides parental migration, are likely to be particularly vulnerable psychologically. Targeted socioemotional support for these children should be prioritized in the migrant-sending communities.

Poor school performance is also strongly associated with children’s total difficulties. In rural China, as in many societies, education is the key vehicle for social mobility and prosperity [38], particularly for rural children who are generally disadvantaged in educational and career opportunities. Consequently, educational achievement is usually a top concern of the child and the entire family, and poorer school performance may increase child’s psychological stress. Lack of parental guidance and supervision in study is likely to worsen such situation.

Our results also showed interesting demographic influences. It is noteworthy that associations of age, sex, and wealth level with psychosocial outcomes differed across multiple dimensions and some even in opposite directions. Future studies should explore the specific mechanisms of these correlations, and develop pertinent care strategies for children across sociodemographic groups.

This study has the following limitations. First, as a cross-sectional study, causal pathways cannot be delineated, for instance, between the migration status and child well-being, and therefore, the findings should be considered exploratory. Second, due to practical constraints in recruiting migrants and lack of literacy in some grandparents, we were unable to collect data from parents and caregivers; thus, all the data are from child self-report, without triangulation of responses from other family members. Third, educational levels of parents and caregivers, and specific indicators of quality of care and relationships, such as the frequency and quality of communication, were not assessed in this study.

Despite the limitations, our study strongly suggests that left-behind children have markedly higher psychosocial difficulties, independent of family circumstances, support, and school performance. In particular, our study points out the lasting negative impact of left-behind experiences, which are often neglected in academic studies, policies, and interventions. Our findings inform the research on LBC globally, by providing a better understanding of the care environment and family structure in the absence of migrant parents. Future studies should more detail on the cyclic pattern of migration, such as duration of separation and reunion and frequency of home visits, and adopt a longitudinal design to provide more in-depth evidence on the long-term impact of parental migration. Specific indicators of relationship dynamics and childcare quality in various family and caregiving contexts should also be further examined.

Other major factors that are associated with psychosocial well-being, especially social support and school performance, require further efforts from local communities to safeguard and promote LBC’s well-being. Current child well-being interventions in China are predominantly focused on economic or material support [39], whereas extreme events such as fatal injuries, rape, and suicide of unsupervised rural children have been frequently reported, including a number of tragedies in Guizhou [40]. Our results can help to inform future strategies and programs in addressing the psychological and developmental risks particularly faced by LBC. In fact, in our study sites, “Children’s Clubs” programs (or others with similar names) have been implemented by the local government and community leadership. In their local village, children can play and study together after school in these clubs, under the care and supervision of volunteers such as local retirees and schoolteachers, as well as university students from nearby cities. These programs offer supervision and support for the LBC using integrated resources at community level, and organize various activities that may benefit child development through social interactions. Future research should explore the effectiveness of similar practices to safeguard child well-being in migrant-sending communities.
